# Meta-analysis of fluoroquinolone antibiotics in the treatment of pelvic inflammatory disease and associated risk factors

**DOI:** 10.1097/MD.0000000000045688

**Published:** 2025-11-28

**Authors:** Leyi Zhang, Yan Wang

**Affiliations:** a Department of Gynaecology, Shengzhou People’s Hospital (Shengzhou Branch of The First Affiliated Hospital of Zhejiang University School of Medicine), Shengzhou, Zhejiang Province, China.

**Keywords:** efficacy, fluoroquinolone antibiotics, meta-analysis, pelvic inflammatory disease, safety

## Abstract

Pelvic inflammatory disease (PID) is a common infectious disease affecting the female reproductive organs and surrounding tissues. Antibiotics remain a cornerstone of current treatment regimens. This work aimed to systematically assess efficacy of fluoroquinolone antibiotics in the treatment of PID through meta-analysis (MA). Randomized controlled trials (RCTs) on fluoroquinolone antibiotics for uncomplicated PID were retrieved from online databases up to July 8, 2024. Statistical analysis was conducted using RevMan5.2, focusing on WBC count, CRP levels, clinical efficacy, and adverse reactions (ARs), with outcomes measured by odds ratios (ORs) and 95% confidence intervals (CIs). Fourteen studies were included, comprising a total of 2483 patients with PID, of whom 1244 received fluoroquinolone antibiotics (experimental group – EG). All patients received treatment for 7 to 14 days. After treatment, the EG showed significantly lower WBC and CRP levels compared to the control group (CG), with both differences statistically significant (*P* < .00001, 5 studies; *I*^2^ = 0% for WBC; *I*^2^ = 0% for CRP). The cure rate in EG was 66.43% (663/998), which was higher than the CG cure rate of 62.42% (623/998) (*P* < .05, 9 studies; *I*^2^ = 42%). The incidence rates of abdominal pain (3.89%, 18/463), nausea and vomiting (17.13%, 168/981), and overall adverse reactions (35.22%, 273/775) were all lower in EG compared to CG (*P* < .05, 7 studies for ARs; *I*^2^ = 54%). Fluoroquinolone antibiotics appear to improve inflammatory markers and may increase the cure rate for PID. Additionally, their incidence of adverse reactions seems lower compared to other treatments. While they show promising efficacy, safety concerns – such as tendon damage, cardiovascular issues, and neuropsychiatric effects – should be carefully considered, especially in at-risk populations. Given these risks and the variability in outcomes, further large-scale, long-term studies are needed to confirm their overall safety and efficacy. Future research should also identify the best patient populations for fluoroquinolone use and explore alternative treatments with comparable benefits and fewer risks.

## 1. Introduction

Pelvic inflammatory disease (PID) is a common infectious disease affecting female reproductive organs and surrounding tissues.^[1–3]^ Common treatments for PID include antibiotics, anti-inflammatory drugs, and analgesics. Anti-inflammatory drugs do not directly eradicate pathogens but play a supportive role in treatment,^[4]^ while analgesics only manage symptoms without curing PID.^[5]^ Therefore, antibiotics remain one of the primary medications for treating PID at present.

Antibiotics used to treat PID primarily include fluoroquinolones, β-lactams, macrolides, aminoglycosides, and trimethoprim derivatives.^[6]^ β-lactam antibiotics provide good coverage against Gram-negative bacteria and certain Gram-positive bacteria, although some Gram-positive bacteria may develop resistance, and some patients may experience allergic reactions.^[7]^ Macrolide antibiotics are effective against Gram-positive bacteria such as streptococci; however, long-term use may lead to resistance development and gastrointestinal adverse effects in some patients.^[8]^ Aminoglycoside antibiotics are typically used as part of combination therapy for more severe or complex infections, but long-term use may cause renal and ototoxicity.^[9]^ Trimethoprim antibiotics exhibit good antibacterial activity against Gram-positive bacteria and Chlamydia, yet they are not suitable for all bacterial types, and some patients may experience allergic reactions. Fluoroquinolone antibiotics are broad-spectrum antimicrobial agents that primarily exert their bactericidal effects by inhibiting bacterial DNA replication and transcription processes. They exhibit wide-ranging coverage against many bacteria. Due to their good tissue penetration and favorable pharmacokinetic properties, fluoroquinolone antibiotics are widely used in the treatment of PID.^[10]^ They are effective in eradicating common pathogens causing PID, such as Salmonella, Escherichia coli, and Streptococcus.^[11]^ However, with prolonged use, concerns regarding the safety of fluoroquinolone antibiotics have been raised. The U.S. Food and Drug Administration (FDA) has issued black box warnings for fluoroquinolones due to the potential risk of serious side effects, including tendinitis, tendon rupture, and peripheral neuropathy. In addition, the use of fluoroquinolones has been associated with an increased risk of serious cardiac arrhythmias, *Clostridioides difficile*-associated diarrhea, and neuropsychiatric effects such as confusion, agitation, and hallucinations. These adverse reactions (ARs) are particularly concerning in older adults and those with underlying health conditions. Although fluoroquinolone antibiotics are effective in the short term for managing PID, these safety concerns warrant careful consideration, especially for long-term or repeated use.^[8]^ While effective in the short term for PID control, long-term use of fluoroquinolone antibiotics may pose safety issues.

Overall, fluoroquinolone antibiotics remain pivotal in the treatment of PID due to their unique antimicrobial mechanisms and superior pharmacokinetic properties. However, their effectiveness and safety profile remain controversial. This work employed meta-analysis (MA) to comprehensively evaluate the outcomes of existing research on fluoroquinolone antibiotic therapy for PID. Through systematic review and quantitative synthesis of the literature, we systematically assessed efficacy and safety of fluoroquinolone antibiotics in PID therapy. Furthermore, it seeks to explore relevant risk factors associated with treating PID and provide guidance for clinical adoptions.

## 2. Data and methodologies

### 2.1. Literature search and screening

This research was approved by the Ethics Committee of Shengzhou People’s Hospital. To obtain all relevant studies concerning fluoroquinolone antibiotic treatment in patients with PID, searches were conducted using the following keywords: “Fluoroquinolone antibiotics,” “Ciprofloxacin,” “Moxifloxacin,” “Levofloxacin,” “levofloxacin hydrochloride,” “Ofloxacin,” “Norfloxacin,” “Enoxacin,” “Trifloxafen,” “Lomefloxacin,” “Perfloxacin,” “Difloxacin” for fluoroquinolone antibiotics, and “pelvic inflammatory disease,” “PID,” “pelvic inflammatory” for PID. Logical operator “or” was used to combine different keywords within fluoroquinolone antibiotics or PID searches, while “and” was used to link fluoroquinolone antibiotics and PID searches. Searches were conducted in Chinese National Knowledge Infrastructure, Wanfang Data, VIP Information, China Biomedical Literature Database, Google Scholar, Medline, Embase, PubMed, Cochrane Library, Nature, Web of Science, Springer, and Science Direct online databases from database inception to July 18, 2024, without language, race, or geographic restrictions. Relevant reference tracking was conducted based on pertinent literature reviews.

### 2.2. Criteria

Inclusion criteria: paper types: randomized controlled trials (RCTs), cohort studies, observational studies, etc; study population: adults aged ≥ 18 years diagnosed with PID based on comprehensive clinical symptoms, physical signs, and laboratory tests, with no restriction on etiology or duration; intervention: experimental groups (EGs) treated with fluoroquinolone antibiotics, control groups (CGs) treated with non-fluoroquinolone antibiotics or other medications; outcome measures: white blood cell count (WBC), C-reactive protein (CRP) levels posttreatment, cure rate, effectiveness rate, incidence of ARs. Cure rate was defined as the proportion of patients who demonstrated complete resolution of symptoms and laboratory signs of infection. Total effectiveness rate, on the other hand, included patients who experienced either full symptom resolution or significant improvement in clinical symptoms, even if they had not achieved a complete cure.

Exclusion criteria: observational studies without a CG, i.e., non-case-control studies; studies where CG received fluoroquinolone antibiotics alone or in combination; studies lacking any of the specified outcome measures; reviews, case reports, or individual pathological studies; studies conducted on animal subjects; duplicate publications.

### 2.3. Screening and data extraction

Two reviewers independently used *EndNote X9* to manage literature for screening. Initially, they screened RCTs on fluoroquinolone antibiotic treatment for PID based on titles and abstracts. They then applied predefined criteria to select studies for full-text review and final inclusion, extracting relevant indicators and data. In cases of disagreement on study inclusion, a third-party expert was consulted for resolution.

Data extraction was performed independently by the reviewers and cross-verified. Extracted information included author details, publication year, study type, sample size, participant age, and outcome measures, all organized in *Excel* for analysis.

### 2.4. Risk assessment of literature bias

Two researchers independently evaluated the quality of literature using the bias risk assessment tool provided by Cochrane *RevMan5.3*, following the evaluation criteria recommended in the Cochrane Handbook. These criteria encompass random sequence generation, allocation concealment, blinding of participants and personnel, blinding of outcome assessors, completeness of outcome data, selective outcome reporting, and other sources of bias. Discrepancies in risk assessment – categorized as “high,” “low,” or “unclear” – were resolved through discussion or consultation with an expert if needed.

### 2.5. Statistical methodologies

MA of the included studies was performed using *RevMan5.3*. Heterogeneity was initially assessed with χ^2^ test at α = 0.05 (*P* < .05). Subsequently, *I*^2^ statistics in *RevMan5.3* were used: *I*^2^ < 50% indicated fixed-effects model (FEM) MA, while *I*^2^ > 50% indicated random-effects model (REM) MA. Forest plots (FOPs) were generated to visualize the results, and *Z*-values and *P*-values from the analysis were used for indicating statistical significance (*P* < .05) with 95% confidence intervals (CIs). Publication bias (PB) was assessed using funnel plots (FUPs) in *RevMan5.3*, followed by Egger linear regression test for quantitative evaluation. A 95% CI including 0 and a *P* > .10 in Egger test indicated no PB. Egger linear regression analysis was conducted using *Stata 10.0*.

## 3. Results

### 3.1. Search results of literature

**Figure 1.**
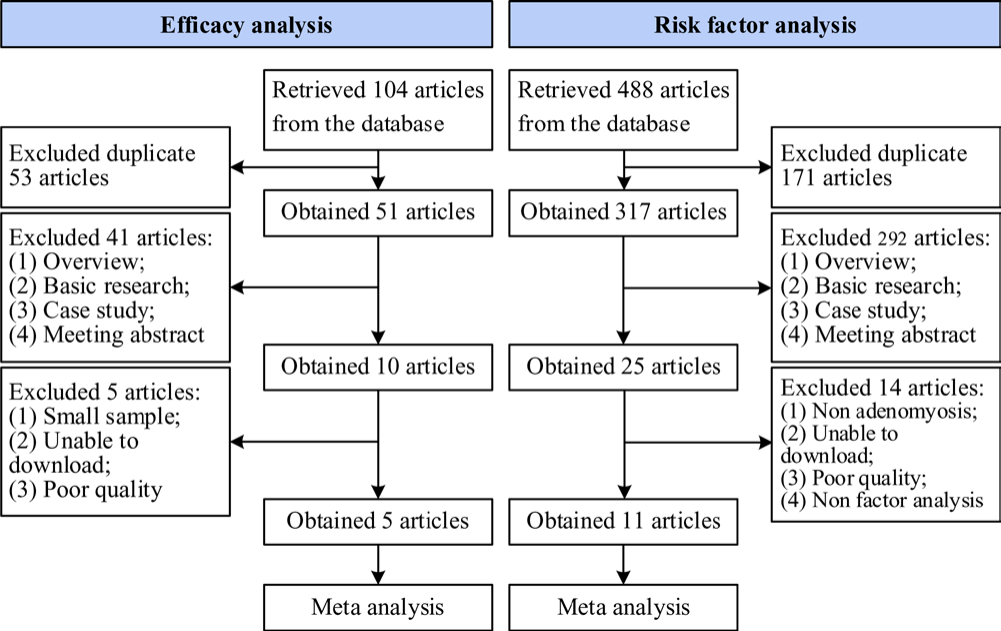
Basic process of literature search.

1450 articles were retrieved from various online databases. After initial screening to exclude 847 review articles, 603 studies remained. Among these, 308 were duplicates and 15 were case reports. After removal of duplicates and case reports, 280 studies remained. Upon further review of abstracts and full texts, 263 studies not meeting inclusion criteria were discarded, leaving 17 studies. Of these, 3 studies were excluded as original data could not be obtained through available channels. Consequently, 14 studies were finally included for analysis. The literature search and screening process for this work are depicted in Figure 1.

### 3.2. Basic information of included literatures

Fourteen published RCTs^[12–25]^ were identified through database searches. All trials adhered to the diagnostic and efficacy assessment criteria specified in the inclusion criteria, with treatment durations ranging from 7 to 14 days. These 14 studies included a total of 2483 patients: 1244 in EG and 1239 in CG. Interventions in EG involved fluoroquinolone antibiotics (Ciprofloxacin, Moxifloxacin, Levofloxacin, Ofloxacin, Perfloxacin) either as monotherapy or in combination with other drugs. CG interventions included drugs such as clindamycin, doxycycline + metronidazole, gentamicin + clindamycin, ceftriaxone + azithromycin + metronidazole, doxycycline + metronidazole, and erythromycin + metronidazole. Neglectable differences existed in patient age, severity of illness, or other demographic characteristics among the groups in all studies. Baseline data including age, gender, distribution of illness, onset time, and microbiological evidence were well-matched across the included literature, ensuring comparability without statistical significance. Summary study information is presented in Table 1.

**Table 1:** Basic information.

First author	year	Country	Research type	Number of cases		Treatment strategy		Treatment time
				EG	CG	EG	CG	
Sha L^[12]^	2020	China	RCT	40	40	Intravenous injection of 0.6 g Lincomycin + 0.2 g Ofloxacin, administered twice daily.	Intravenous injection of 0.6 g of Clindamycin hydrochloride, administered twice daily.	14 d
Heystek M^[13]^	2009	UK	Prospective double-blind RCT	343	326	400 mg moxifloxacin per day	100 mg doxycycline [twice daily] + 400 mg metronidazole [three times daily]	14 d
Apuzzio JJ^[14]^	1989	USA	RCT	48	46	30 mg of Ciprofloxacin, administered twice daily.	1.5 mg/kg of Gentamicin, administered 3 times daily, plus 900 mg of Clindamycin, also administered 3 times daily.	14 d
Dean G^[15]^	2021	UK	RCT	153	160	400 mg of Ofloxacin combined with 400 mg of Metronidazole	500 mg of Ceftriaxone administered intramuscularly, combined with Azithromycin treatment (1 g on the first day followed by 500 mg daily for days 2–5), and 700 mg of Metronidazole administered twice daily.	14 d
Arredondo JL^[16]^	1997	USA	Double-blind RCT	69	69	30 mg of Clindamycin (administered 3 times daily) combined with 250 mg of Ciprofloxacin (administered twice daily).	250 mg of Ceftriaxone administered intramuscularly, combined with 100 mg of Doxycycline (administered twice daily).	14 d
Witte EH^[17]^	1993	Netherlands	RCT	20	20	800 mg of Pefloxacin (administered 13 times daily) combined with 500 mg of Metronidazole (administered 3 times daily).	100–200 mg of Doxycycline combined with 500 mg of Metronidazole (administered 3 times daily).	14 d
Martens MG^[18]^	1993	USA	Prospective RCT	138	134	400mg Ofloxacin (administered twice daily).	2 g of Ceftriaxone, administered with 1 g of Probenecid, and 100 mg of Doxycycline twice daily.	10 d
Priyadharshini M^[19]^	2019	India	RCT	30	30	400 mg of Moxifloxacin once daily.	100 mg of Doxycycline and 500 mg of Metronidazole administered twice daily.	14 d
De R^[20]^	2020	India	Prospective double-blind RCT	50	50	500 mg Ofloxacin	100 mg of Erythromycin combined with 500 mg of Metronidazole	14 d
Moghtadaei P^[21]^	2008	Iran	RCT	70	80	250 mg Ceftriaxone + 200 mg Ofloxacin (administered once daily).	250 mg of Ceftriaxone administered once daily, combined with 1 g of Azithromycin administered once weekly.	14 d
Sharma JB^[22]^	2007	India	Prospective RCT	98	95	400 mg of Ofloxacin, 500 mg of Metronidazole, 10 mg of Serratiopeptidase, along with 60 million units of Lactic acid bacillus and 2 million units of Saccharomyces boulardii.	100 mg doxycycline + 400 mg metronidazole + 10 mg serratiopeptidase	14 d
Mirblook F^[23]^	2011	Iran	RCT	93	96	400 mg Ofloxacin + 500 mg Metronidazole	1 g Azithromycin + 500 mg Metronidazole	14 d
Malhotra M^[24]^	2003	India	RCT	52	53	500 mg ciprofloxacin + 600 mg tinidazole, (administered twice daily).	100 mg of Doxycycline administered twice daily, combined with 200 mg of Metronidazole administered twice daily.	7 d
Wang X^[25]^	2017	China	RCT	40	40	0.5 g of Metronidazole administered twice daily, combined with 0.1 g of Ofloxacin administered twice daily.	0.5 g of Metronidazole administered twice daily.	10 d

### 3.3. Literature quality evaluation

**Figure 2.**
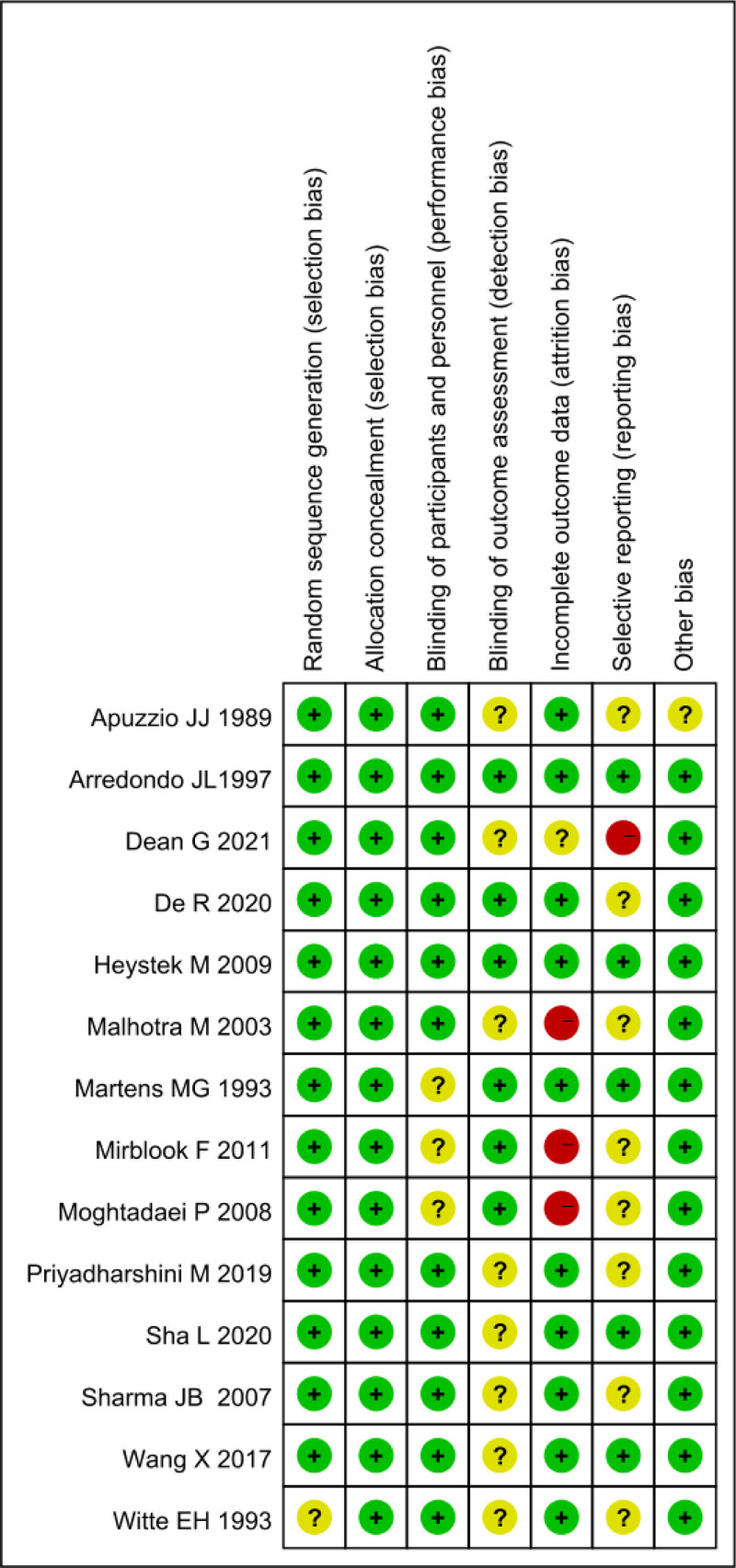
Risk bias assessment for included studies.

In this work, 14 RCTs were included. Among them, 13 studies described their methods of randomization, implying low risk of bias. One study mentioned randomization but did not specify the method, thus bias risk could not be assessed. Only 3 studies among those included mentioned the use of double-blinding and provided details on its implementation, while the remaining eleven studies did not describe the process of concealing the random sequence, precluding assessment of related bias risks. Regarding outcome reporting, 5 studies fully reported their outcome data, while the others had varying degrees of missing data. However, without access to the study protocols, selective outcome reporting bias could not be determined, and assessment of other sources of bias was also not possible. Details of bias risk assessments for the included studies are provided in Figure 2.

### 3.4. WBC

**Figure 3.**
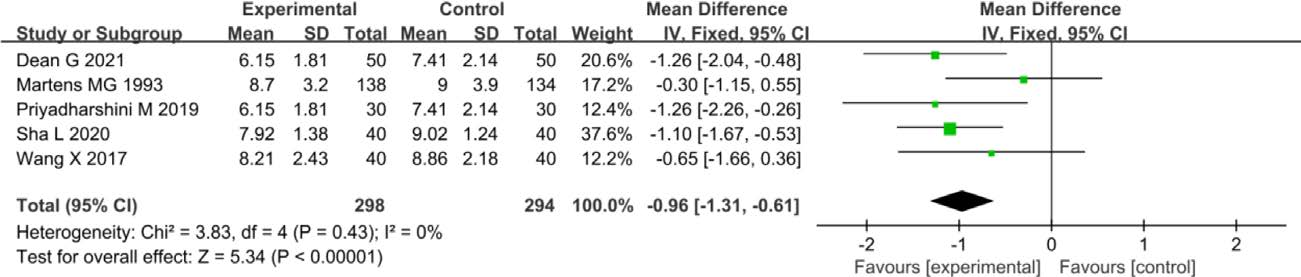
FOP of WBC analysis after treatment. FOP = forest plot, WBC = white blood cell.

Five studies included in the analysis provided posttreatment WBC values for both EG and CG. Heterogeneity testing among these studies yielded an *I*^2^ value of 0% and a *P*-value of 0.43, implying homogeneity among the WBC measurements across studies. Hence, a FEM was employed for the MA. The pooled effect size (OR) across the 5 studies was −0.96 (95% CI: −1.31 to −0.61), with a test of significance yielding *Z* = 5.34 and *P* < .00001. Thus, the WBC levels after treatment were markedly lower in EG versus CG (*P* < .00001) (Fig. 3).

**Figure 4.**
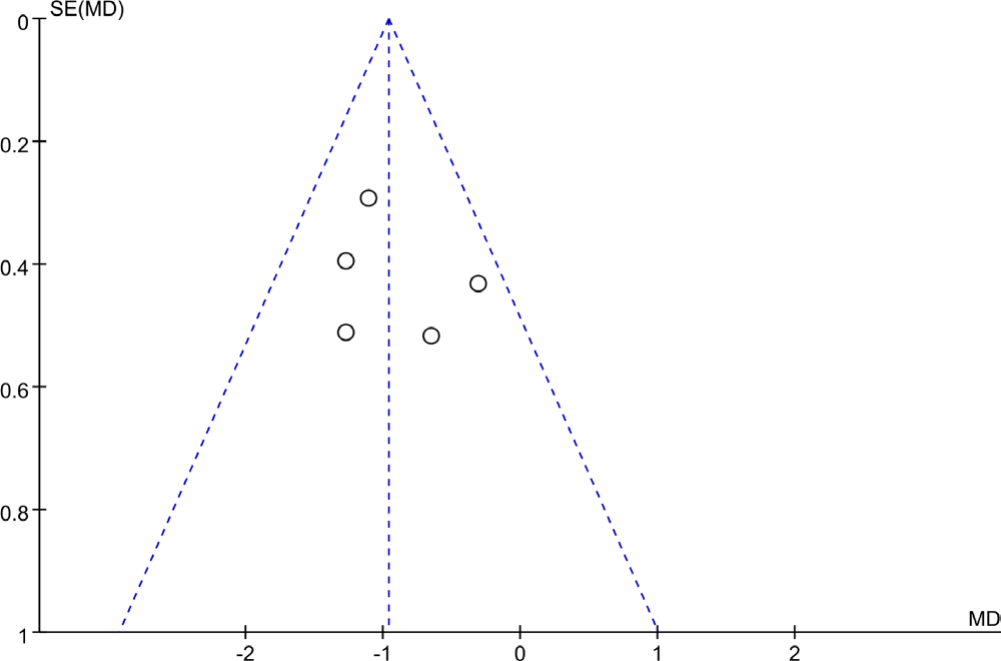
FUP of WBC analysis after treatment. FUP = funnel plot, WBC = white blood cell.

A FUP was utilized to analyze PB in the assessment of posttreatment WBC levels across the included studies. In Figure 4, the scatter distribution on the FUP was symmetrical, with all points falling within the confines of the funnel, suggesting that there was neglectable PB.

### 3.5. CRP

**Figure 5.**
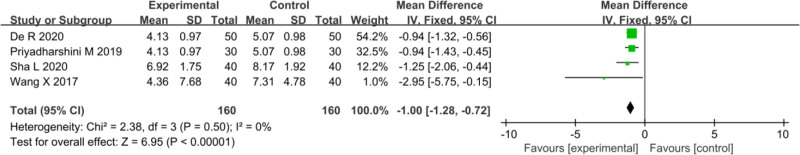
FOP of CRP analysis after treatment. CRP = C-reactive protein, FOP = forest plot.

Four studies included in the analysis provided posttreatment CRP values for both EG and CG. Heterogeneity testing among the studies yielded *I*^2^ = 0 and *P* = .50, implying homogeneity in CRP levels across the studies. Therefore, a FEM was employed for MA, yielding an overall effect size OR of −1.00 (95% CI: −1.28 to −0.72), with a marked *Z*-test result of 6.95 (*P* < .00001). Hence, the CRP levels posttreatment were lower in EG versus CG (*P* < .00001) (Fig. 5).

**Figure 6.**
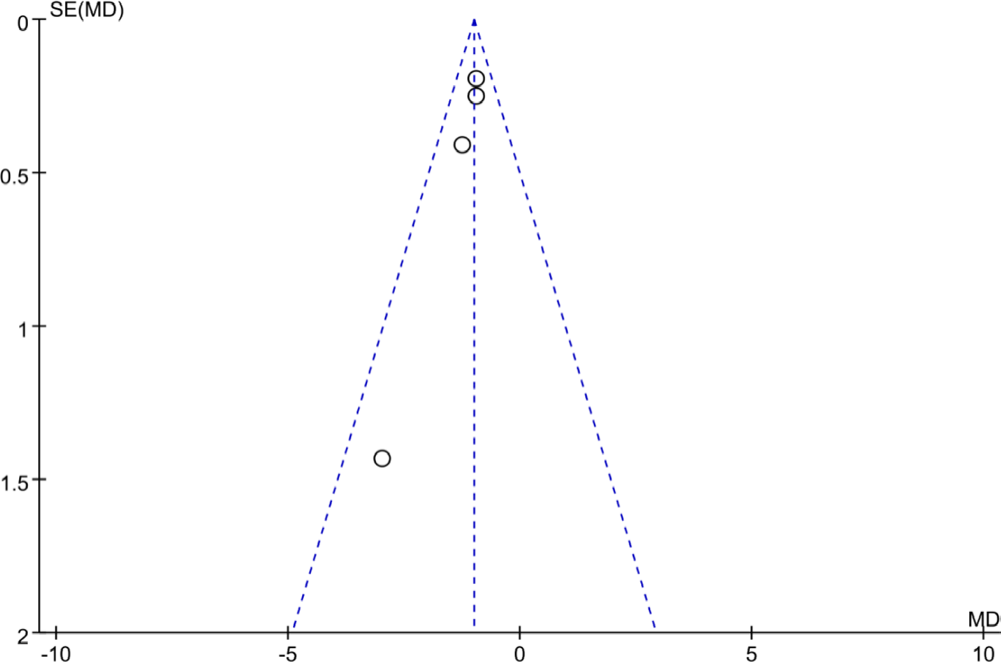
FUP of CRP analysis after treatment. CRP = C-reactive protein, FUP = funnel plot.

A FUP was used to analyze PB in the included literature on posttreatment CRP levels. In Figure 6, the scatter distribution in the FUP was symmetrical, and all points lied within the funnel, implying inconsiderable PB.

### 3.6. Clinical efficacy

**Figure 7.**
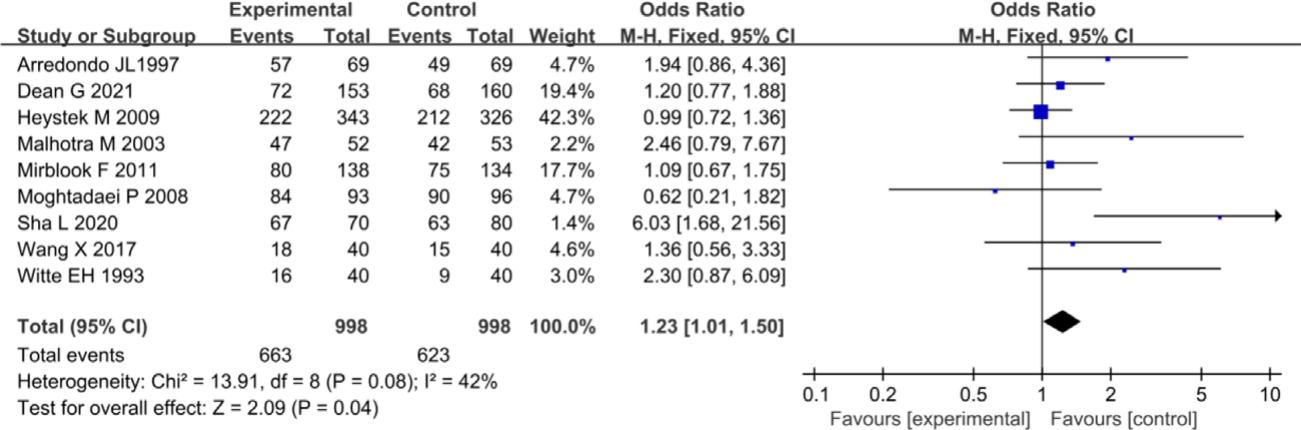
FOP of cure rate analysis. FOP = forest plot.

Nine studies included in the analysis provided data on cure rates and PID cure rates for both the treatment and CGs. Heterogeneity testing among these studies yielded an *I*^2^ value of 42% and a *P*-value of 0.08, implying homogeneity in the cure rates across studies. Hence, a FEM was employed for MA, yielding an overall OR of 1.23 (95% CI: 1.01–1.50). The test for significance resulted in *Z* = 2.09, with *P* = .04. The cure rate in the treatment group was 66.43% (663/998), which was higher than CG’s 62.42% (623/998) (*P* < .05) (Fig. 7).

**Figure 8.**
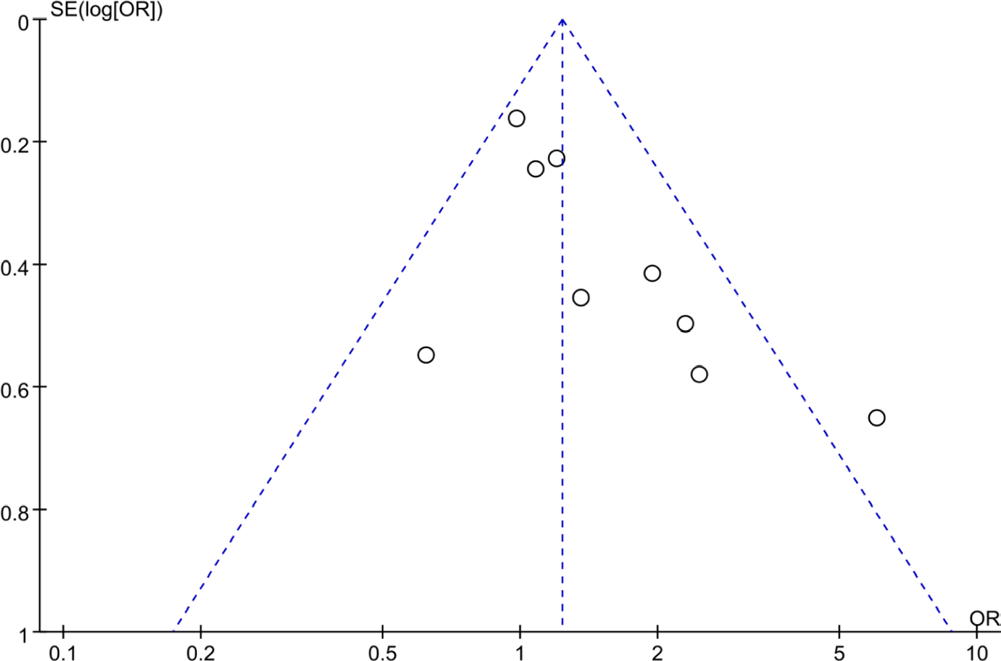
FUP of cure rate analysis. FUP = funnel plot.

Using a FUP, we analyzed PB in the included literature regarding cure rates. As depicted in Figure 8, the scatter distribution on the FUP was symmetrical, with most studies falling within the funnel, implying slight PB.

**Figure 9.**
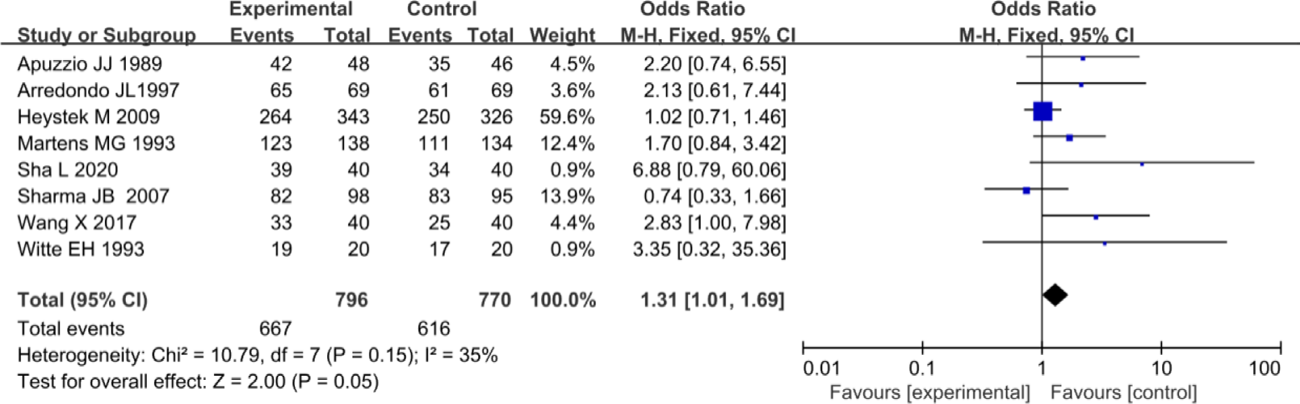
FOP of treatment effectiveness analysis. FOP = forest plot.

In the included studies, 8 trials provided data on the overall treatment efficacy rates of PID patients in both EG and CG. Heterogeneity testing among these studies showed *I*^2^ = 35%, *P* = .15, implying homogeneity in treatment efficacy rates among the studies. Therefore, a FEM was employed for the MA, yielding an overall effect size OR of 1.31 (95%CI: 1.01–1.69), with a considerable test result of *Z* = 2.00, *P* = .05. Thus, slight difference existed in treatment efficacy rates between EG and CG for PID patients (*P* = .05) (Fig. 9).

**Figure 10.**
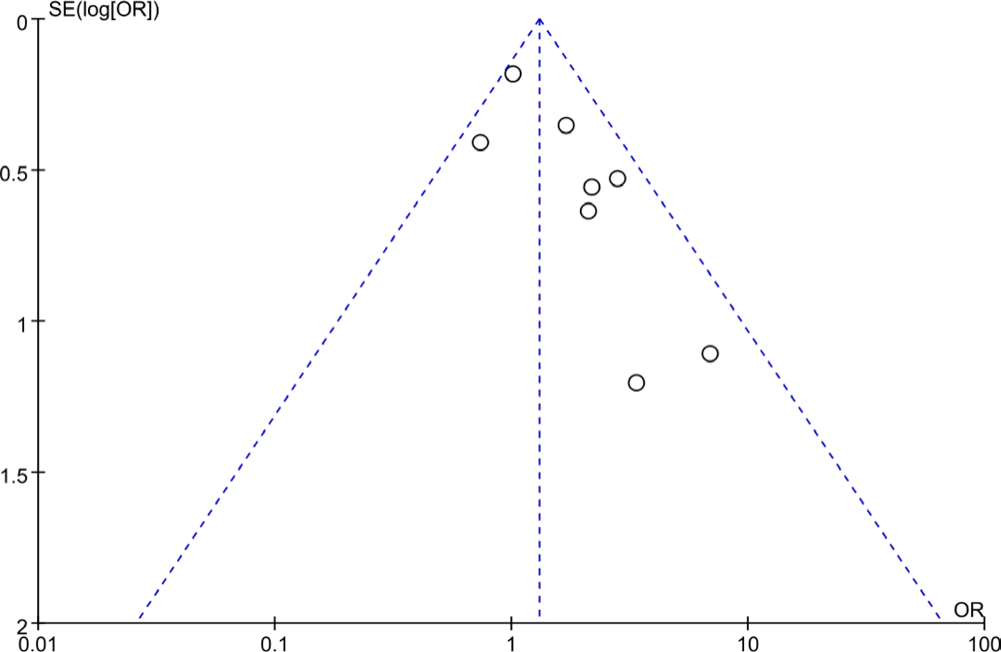
FUP of treatment efficacy analysis. FUP = funnel plot.

Using a FUP, PB was assessed for the analysis of treatment effectiveness rate from the included literature. Figure 10 illustrates that the scatter distribution on the FUP was symmetric, and all studies fell within the funnel, implying slight PB.

### 3.7. Adverse reactions

**Figure 11.**
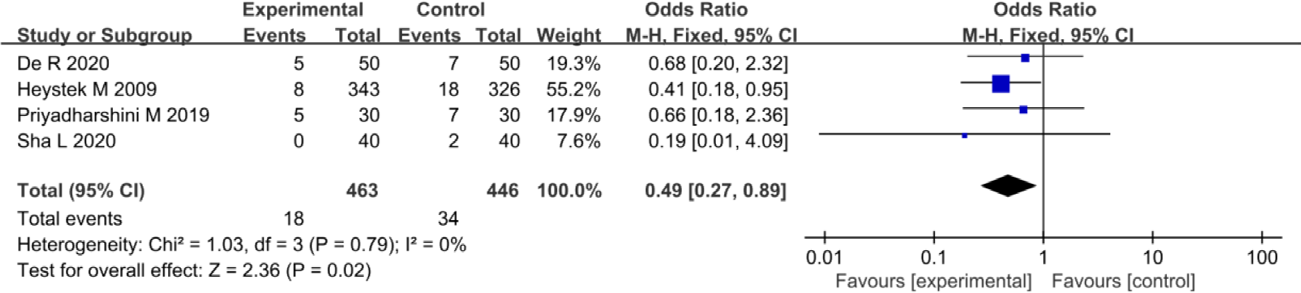
FOP of abdominal pain incidence rate. FOP = forest plot.

In the included studies, 4 trials provided data on the occurrence of posttreatment abdominal pain in PID patients between EG and CG. Heterogeneity testing among these studies yielded *I*^2^ = 0, *P* = .79, implying homogeneity in posttreatment abdominal pain rates across the studies. Hence, a FEM was employed for MA, yielding an overall OR of 0.49 (95% CI: 0.27–0.89). The statistical test showed *Z* = 2.36, *P* = .02, implying a notable difference. The incidence of posttreatment abdominal pain in PID patients was 3.89% (18/463) in EG, which was inferior to the 7.62% (34/446) observed in CG. Hence, the incidence of posttreatment abdominal pain in PID patients was markedly lower in EG versus CG (*P* < .05) (Fig. 11).

**Figure 12.**
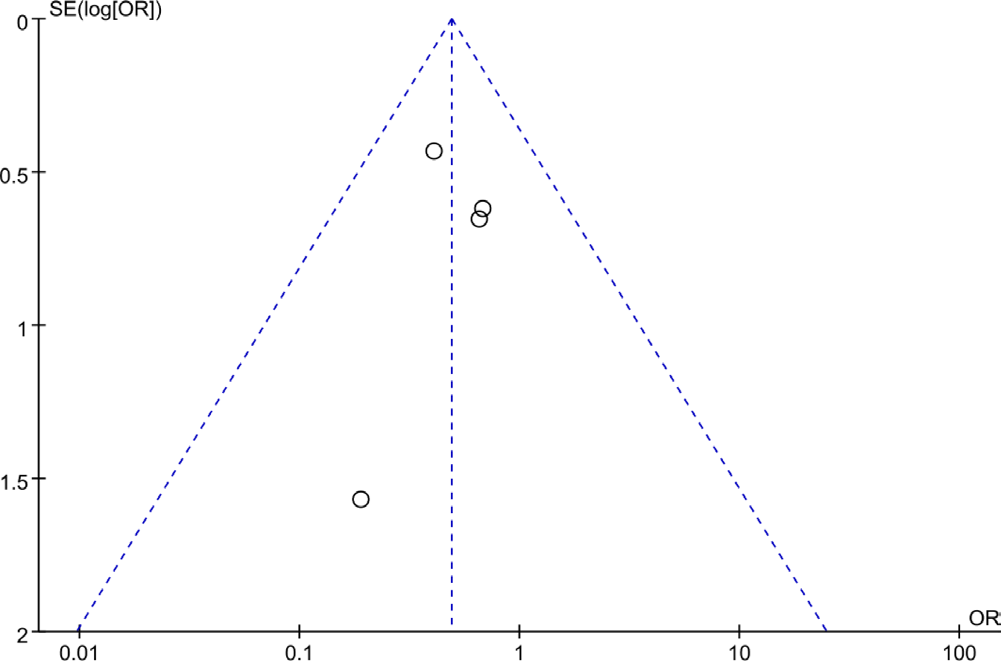
FUP of analysis of abdominal pain incidence rate. FUP = funnel plot.

The PB of posttreatment abdominal pain incidence in PID patients was assessed using a FUP analysis (Fig. 12). The scatter distribution of the FUP was symmetrical, and all studies were within the FUP, implying no marked PB.

**Figure 13.**
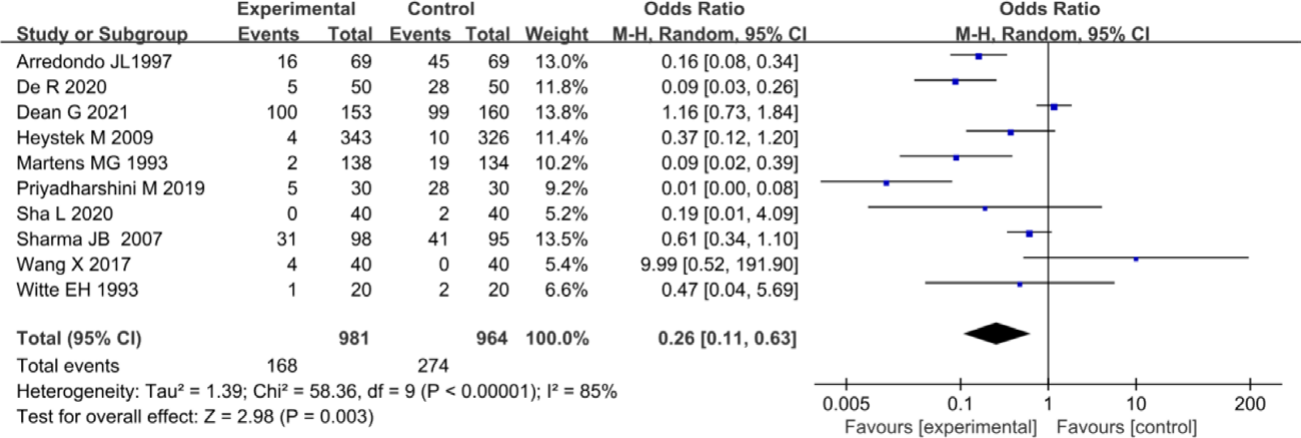
FOP of nausea and vomiting incidence rate. FOP = forest plot.

In the included studies, 10 studies provided data on the incidence of posttreatment nausea and vomiting in PID patients in both EG and CG. Heterogeneity test suggested great heterogeneity across studies (*I*^2^ = 85%, *P* < .00001). Hence, a REM was used for MA. The pooled OR for posttreatment nausea and vomiting incidence across studies was 0.26 (95% CI: 0.11–0.63), with a notable test result of *Z* = 2.98, *P* = .003. The incidence of posttreatment nausea and vomiting was 17.13% (168/981) in EG and 28.42% (274/964) in CG. Thus, PID patients in EG had a lower incidence of posttreatment nausea and vomiting versus CG (*P* < .05) (Fig. 13).

**Figure 14.**
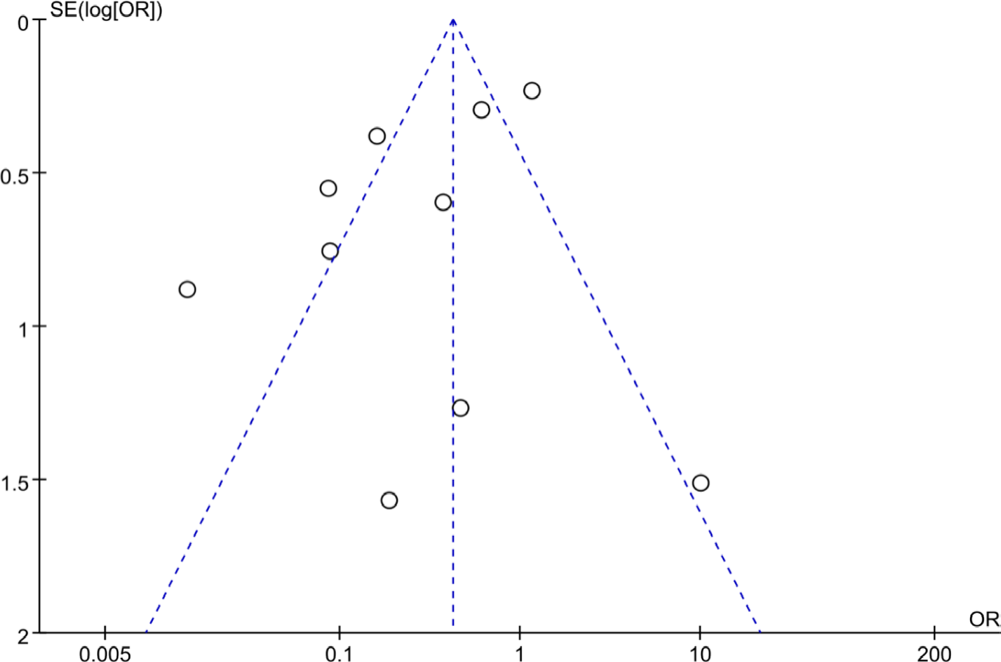
FUP of analysis of nausea and vomiting incidence rate. FUP = funnel plot.

Using a FUP, PB in incidence of nausea and vomiting after treatment in PID patients was analyzed based on the included literature. In Figure 14, the FUP exhibited an asymmetric scatter distribution, with some studies lying outside the funnel, implying a certain degree of PB among the studies.

**Figure 15.**
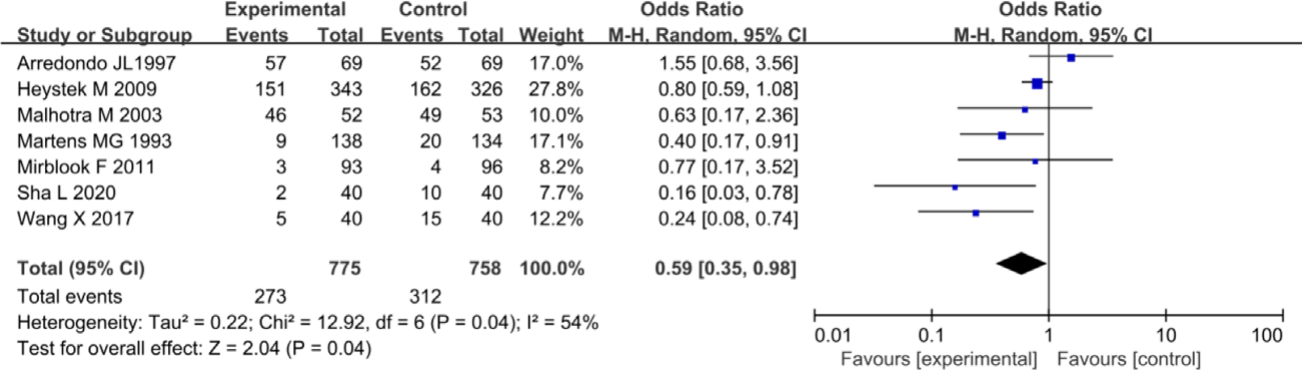
FOP of overall incidence of ARs. ARs = adverse reactions, FOP = forest plot.

In the included studies, 7 trials provided data on the overall incidence of ARs after treatment in PID patients in both EG and CG. Heterogeneity testing among the studies showed *I*^2^ = 54%, *P* = .004, implying marked heterogeneity in the overall incidence of ARs after treatment. Therefore, a REM was used for the MA. The combined effect size OR for the overall incidence of ARs after treatment across the studies was 0.59 (95% CI: 0.35–0.98), with a notable test result of *Z* = 2.04, *P* = .04. The overall incidence of ARs after treatment was 35.22% (273/775) in EG and 41.16% (312/758) in CG, implying a lower incidence in EG versus CG (*P* < .05) (Fig. 15).

**Figure 16.**
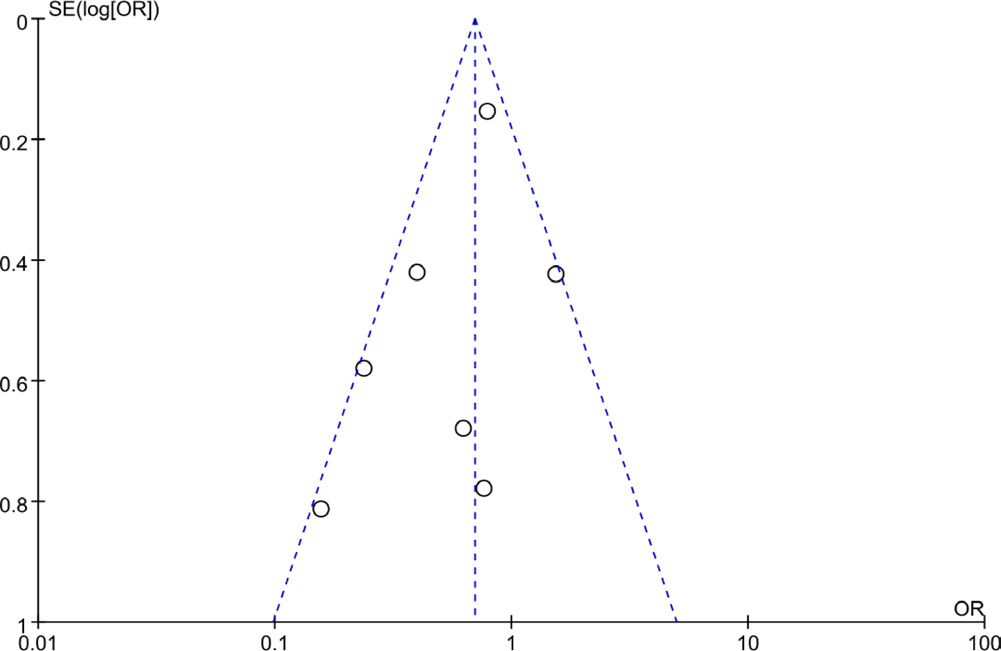
FUP of overall AR incidence analysis. AR = adverse reaction, FUP = funnel plot.

Using a FUP, we analyzed PB in the MA of the overall incidence of ARs after treatment in PID patients. In Figure 16, the scatter plot distribution in the FUP was largely symmetrical, and all included studies were within the bounds of the FUP, implying neglectable PB among the studies.

## 4. Discussion

This work employed MA to assess efficacy of fluoroquinolone antibiotics in treating PID. The results indicated that fluoroquinolone antibiotics markedly improved inflammatory markers and increased the cure rate after treating PID, while greatly reducing the incidence of ARs. These findings suggested that fluoroquinolone antibiotics have great clinical efficacy and good safety profiles in treating PID. These discoveries highlight fluoroquinolone antibiotics as an effective option for treating PID, particularly in cases requiring rapid infection control and symptom reduction. The use of fluoroquinolone antibiotics can decrease patient discomfort and the risk of complications during treatment, thereby enhancing treatment safety and providing a basis for developing more scientific therapeutic strategies.

The primary cause of PID is bacterial ascent from the vaginal and cervical mucosa or via the lymphatic system. Additionally, some cases result from the spread of inflammation from adjacent organs (e.g., appendicitis) or hematogenous dissemination.^[26]^ Common pathogens associated with PID include *Staphylococcus*, *Streptococcus*, *Escherichia coli*, anaerobes, as well as sexually transmitted pathogens such as *Neisseria gonorrhoeae*, *Chlamydia trachomatis*, and *Mycoplasma genitalium*.^[27]^ Prompt initiation of standard antibiotic therapy is crucial once acute PID occurs. Fluoroquinolone antibiotics are known for their potent antibacterial effects and broad-spectrum coverage against pathogens commonly implicated in PID. However, the applicability of these antibiotics may vary across different regions due to differences in drug resistance patterns and regional treatment guidelines. In areas with higher fluoroquinolone resistance, their efficacy in treating PID may be reduced, and local treatment guidelines may recommend alternative therapies. Therefore, while fluoroquinolone antibiotics show promise in treating PID in some regions, their use should be considered in the context of local resistance data and clinical guidelines.^[28]^ This MA included 2483 PID patients and systematically compared the clinical efficacy and safety of fluoroquinolone antibiotics versus non-fluoroquinolone antibiotics in treating PID. The study results indicated that after treatment with fluoroquinolone antibiotics, PID patients showed lower levels of WBC and CRP versus those treated with non-fluoroquinolone antibiotics. This outcome suggests that fluoroquinolone antibiotics effectively reduce inflammatory markers in PID patients. Fluoroquinolone antibiotics possess potent anti-inflammatory effects and can effectively eradicate sources of PID infection,^[29]^ thereby notably reducing inflammatory responses and improving inflammatory markers. These antibiotics exhibit good distribution and tissue penetration in the body, particularly achieving high drug concentrations in the pelvic region,^[30]^ which enhances their bactericidal effects against pathogens and effectively alleviates infection-related inflammation. Fluoroquinolone antibiotics exert strong inhibitory effects on inflammation by interfering with bacterial DNA replication and transcription, thereby effectively reducing inflammatory markers.^[31]^ In contrast, non-fluoroquinolone antibiotics may not demonstrate comparable efficacy in this regard. Multiple studies also demonstrated that fluoroquinolone antibiotics greatly improve inflammatory markers in the treatment of PID,^[32–35]^ consistent with our study findings. Regarding clinical efficacy, our study results showed a cure rate of 66.43% (663/998) in EG, which was superior to the 62.42% (623/998) in CG, but the treatment effectiveness differed slightly versus CG. This indicates that fluoroquinolone antibiotics perform better in terms of cure rate versus non-fluoroquinolone antibiotics, but did not significantly outperform them in treatment effectiveness. This difference may be due to various complex factors, including drug selection, study design, sample selection, or treatment regimens. Future research should further explore these factors to gain a deeper understanding of the practical effects and clinical guidance of different treatment strategies in PID management.

Fluoroquinolone antibiotics, such as moxifloxacin and levofloxacin, are commonly used for treating PID due to their strong antimicrobial efficacy and relatively low incidence of ARs compared to other antibiotic classes. However, despite these benefits, fluoroquinolones are associated with several potentially serious ARs. These include tendon damage (e.g., tendinitis and tendon rupture), which can be especially concerning in older adults and those using corticosteroids. Additionally, fluoroquinolones can cause prolonged QT intervals, leading to an increased risk of cardiac arrhythmias, particularly in patients with underlying heart conditions. Neurological reactions such as seizures, headaches, dizziness, insomnia, fatigue, and peripheral neuropathy have also been reported. These adverse effects highlight the need for cautious use, particularly in high-risk populations. Despite these risks, fluoroquinolones generally exhibit lower rates of gastrointestinal symptoms, such as nausea, vomiting, and diarrhea, as well as musculoskeletal symptoms like muscle spasms and joint pain, compared to other antibiotics.^[36–38]^ The study results indicated that the occurrence rates of abdominal pain, nausea/vomiting, and overall ARs in PID patients after treatment in EG were 3.89%, 17.13%, and 35.22%, respectively, all of which were inferior to those in CG. This suggests that fluoroquinolone antibiotics exhibit better tolerance and safety profiles in the treatment of PID versus non-fluoroquinolone antibiotics. Fluoroquinolone antibiotics exert their effects by interfering with bacterial DNA synthesis and replication.^[39]^ This mechanism may result in lower irritability to intestinal tissues versus certain other antibiotics, thereby reducing the incidence of gastrointestinal ARs in patients during usage. In contrast, some non-fluoroquinolone antibiotics may induce more gastrointestinal discomfort and other ARs, possibly due to their pharmacological mechanisms and metabolic pathways. Similar findings also demonstrated lower incidence rates of ARs with fluoroquinolone antibiotics in the treatment of PID.^[40,41]^ These findings align with our study results, further supporting the clinical advantages of these drugs. The use of fluoroquinolone drugs is also associated with increased risks of superficial cardiac conduction abnormalities and other cardiac issues, central nervous system symptoms, and glucose abnormalities (especially in diabetic patients).^[42,43]^ Additionally, long-term or excessive use of fluoroquinolone drugs may lead to severe muscle pain or even rhabdomyolysis,^[44]^ a condition known as fluoroquinolone-associated muscle pain or tendon injury. However, specific studies have not statistically analyzed these ARs, thus comparisons regarding the occurrence of these ARs were not possible.

While this work yielded positive results regarding fluoroquinolone antibiotics in PID treatment, several limitations must be considered. First, there was heterogeneity in the study populations, including differences in age, disease severity, and underlying conditions. Additionally, diverse treatment regimens across the included studies may have influenced outcomes. Incomplete reporting of ARs further limited the ability to assess safety comprehensively. The quality of the studies varied, with some lacking details on randomization, blinding, and statistical analysis, which could introduce bias. Moreover, PB and missing data in some studies may have impacted the robustness of the findings. In conclusion, although this MA demonstrates the potential of fluoroquinolone antibiotics for treating PID, further research with more rigorous methodology, larger sample sizes, and better reporting is needed to confirm these findings and guide clinical practice.

## 5. Conclusion

This work evaluated the efficacy and safety of fluoroquinolone antibiotics in treating PID. A systematic search across various databases identified 14 studies for analysis. The results indicated that fluoroquinolone antibiotics significantly reduced WBC and CRP levels in PID patients, increased the cure rate, and lowered the occurrence of abdominal pain, nausea/vomiting, and overall ARs. This suggests that fluoroquinolone antibiotics effectively alleviate inflammatory responses and demonstrate significant clinical efficacy and safety in treating PID. The study provides strong support and scientific evidence for the clinical application of fluoroquinolone antibiotics in PID treatment, which is crucial for guiding clinical practice and future working directions.

## Author contributions

**Conceptualization:** Leyi Zhang, Yan Wang.

**Data curation:** Leyi Zhang, Yan Wang.

**Formal analysis:** Leyi Zhang, Yan Wang.

**Investigation:** Leyi Zhang, Yan Wang.

**Methodology:** Leyi Zhang, Yan Wang.

**Project administration:** Yan Wang.

**Supervision:** Leyi Zhang, Yan Wang.

**Validation:** Yan Wang.

**Visualization:** Leyi Zhang, Yan Wang.

**Writing – original draft:** Leyi Zhang, Yan Wang.

**Writing – review & editing:** Leyi Zhang, Yan Wang.
